# Instructed versus spontaneous entrainment of running cadence to music tempo

**DOI:** 10.1111/nyas.14528

**Published:** 2020-11-18

**Authors:** Edith Van Dyck, Jeska Buhmann, Valerio Lorenzoni

**Affiliations:** ^1^ IPEM Ghent University Ghent Belgium

**Keywords:** music, running, movement, entrainment, auditory–motor coupling

## Abstract

Matching exercise behavior to musical beats has been shown to favorably affect repetitive endurance tasks. In this study, our aim was to explore the role of spontaneous versus instructed entrainment, focusing on self‐paced exercise of healthy, recreational runners. For three 4‐min running tasks, 33 recreational participants were either running in silence or with music; when running with music, either no instructions were given to entrain to the music, or participants were instructed to match their running cadence with the tempo of the music. The results indicated that less entrainment occurred when no instruction to match the exercise with the musical tempo was provided. In addition, similar to the condition without music, lower speeds and shorter step lengths were observed when runners were instructed to match their running behavior to the musical tempo when compared with the condition without such instruction. Our findings demonstrate the impact of instruction on running performance and stress the importance of intention to entrain running behavior to musical beats.

## Graphical abstract

In this study, our aim was to explore the role of spontaneous versus instructed entrainment, focusing on self‐paced exercise of healthy, recreational runners. Results revealed that less entrainment occurred when no instruction to match the exercise with the musical tempo was provided. In addition, similar to the condition without music, lower speeds and shorter step lengths were observed when runners were instructed to match their running behavior to the musical tempo when compared with the condition without such instruction.

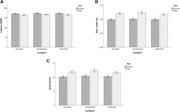

## Introduction

Music and exercise—the combination of the two is often regarded as a great match, a viewpoint that has important implications for the sports and exercise domain. It is, for instance, hard to imagine a gym deprived of loud, energetic music motivated by the conviction that music boosts performance. In sports and exercise research, this hypothesis has been tested repeatedly, demonstrating that music is indeed capable of increasing exercise intensity and endurance,^1^, ^2^, ^3^, ^4^, ^5^ stimulating rhythmic movement,^4^ distracting from fatigue and discomfort,^6^, ^7^ prompting and altering mood states,^7^ spurring motivation,^8^ inducing arousal,^9^ relieving stress,^6^ and evoking a sense of power and producing power‐related cognition and behavior.^10^ Music to which performance can be synchronized in particular was shown to extend endurance and increase exercise intensity.^11^

The process underlying this particular type of auditory–motor coupling is commonly referred to as *entrainment*. It entails a match of musical tempi with exercising tempi, locked in a particular period relationship (e.g., running tempo matching musical tempo) and resulting in regular corporeal patterns. Performance boosting effects of entrained music are rooted in the ability of motor‐to‐music entrainment to reduce the metabolic cost of exercise by enhancing neuromuscular or metabolic efficiency.^12^, ^13^ Owing to the absence of timely adjustments within the kinetic pattern and an increase in the level of relaxation resulting from the precise expectancy of the forthcoming movement, regular corporeal patterns demand less energy to imitate.^14^ Hence, by employing music that can be corporeally emulated, a point of reference is established that is able to attract and thus entrain recurring motor patterns.^13^, ^15^

Entrainment and its related benefits were shown to be particularly useful for repetitive endurance tasks, such as walking, running, rowing, and cycling.^11^, ^12^, ^16^, ^17^, ^18^ In addition, improvements in endurance performance proved to be most apparent at low‐to‐moderate exercise intensities.^6^, ^19^ This is largely explained by Rejeski's parallel processing hypothesis, which states that as exercise intensity increases, physiological cues (e.g., heart and respiration rates) predominate.^20^ Thus, when the exercise becomes too strenuous, perception of neural exertion signals coming from the muscles, joints, and cardiopulmonary systems increases, resulting in an attention shift toward the painful and/or fatiguing effects of the exercise.^20^, ^21^, ^22^, ^23^, ^24^

Most previous research on the effects of music‐to‐motor coupling on exercise and sports focused on instructed (or imposed, intended) entrainment (e.g., see Refs. 9, 11, 16, 25, and 26). In this case, the exerciser is explicitly instructed to match his/her exercise behavior to a musical beat or pulse. However, entrainment can also occur spontaneously, or when the exerciser is not instructed to match his/her behavior to the music. Although less research has been performed regarding spontaneous (or uninstructed/unintended) entrainment, some have indicated that humans indeed possess a natural predisposition to respond to rhythmical qualities of music.^27^, ^28^, ^29^ Yet, spontaneous entrainment of one tempo with another is only believed to occur when the strength of the coupling is able to overcome possible contrasts in the natural movement period or tempo. The difference between the period of the music and that of the exercise, thus, should not exceed a specific range, referred to as the *entrainment basin*.^28^, ^30^, ^31^, ^32^

It remains rather unclear whether these different approaches could result in divergent effects on performance output, as research combining both instructed and spontaneous entrainment is sparse. However, some research on walking behavior did compare both approaches, stressing the limitations of spontaneous entrainment.^29^, ^33^ Moreover, when the required intensity to match the walking behavior to the beats proved too large, it was shown that intentional entrainment with an active cognitive control mechanism was required in order to obtain movement‐to‐music coupling.^29^

In our study, the aim was to further explore possible differences between instructed and spontaneous entrainment by focusing on a repetitive endurance exercise, namely running. We used a within‐subjects design to investigate possible contrasts in the effects of both approaches (complemented with a baseline condition without music, serving as a point of reference) on a selection of key outcome measures. As music was indicated to be of greater benefit to untrained or recreationally‐active individuals than to those who are highly trained,^34^, ^35^ and since this group is heavily represented in current society, recreational runners were targeted here. Intrinsically, the goal of our study was to provide outcomes that might prove to be of interest to a large population of exercisers and valuable to future research on music and exercise.

## Method

### Participants

To establish sample size, power analysis for a repeated‐measures design was conducted using G^*^Power 3.1.9.2.^36^ On the basis of a small effect size, with alpha set at 0.05 and power at 0.90, it was estimated that about 32 participants would be required. Thirty‐three healthy adult participants (18 females/15 males) took part in the study. The test group consisted of recreational runners with an average age of 34.21 years (SD = 8.17), a mean body mass of 62.49 kg (SD = 12.95), and an average height of 1.70 m (SD = 0.11), who reported being fit enough to run comfortably for at least 30 min without feeling exhausted. Only a minority (36.36%) had received musical training. On average, musically trained participants had 9.50 years (SD = 12.03) of musical experience and were educated in music schools (37.50%) or conservatories (6.25%), through private lessons (37.50%), self‐education (18.75%), or a combination of the above. All participants reported running regularly, with varying degrees of frequency (66.67% reported running multiple times a week; 30.30% about once a week; and 3.03% about once a month). Of all participants, 51.52% reported generally running without music, 39.39% typically trained with music, and 9.09% ran both with and without musical accompaniment. Fisher's exact test showed no significant association between participants’ sex and their musical background (*χ* ^2^(1) = 0.16, *P* = 0.73) or their habit to run to music (*χ* ^2^(2) = 0.92, *P* = 0.69).

### Ethics statement

The study was approved by the Ethics Committee of the Faculty of Arts and Philosophy at Ghent University, Belgium, and all procedures followed were in accordance with the Declaration of Helsinki. In addition, all participants signed a form to declare that they participated voluntarily; that they had received sufficient information concerning the tasks, procedures, and technologies used; that they had the opportunity to ask questions; and that they were aware of the fact that running movements were measured for scientific and educational purposes only.

### Stimulus

For all participants and conditions, the same music track was played to control for possible effects of musical characteristics. However, since recreational running tempi generally vary between 130 and 200 steps per minute (SPM), the track was required to efficiently deal with substantial tempo variations. Yet, to minimize the degree of tempo‐stretching, a stimulus with an original tempo of about 165 beats per minute (BPM) was selected. Furthermore, to facilitate the activating character, clearly audible beats were mandatory as a stable tempo throughout the entire track.^37^ Finally, to further facilitate the imperceptibility of the tempo‐stretching, a track was selected with low to no appearances in national and international music charts, that is, one that was unfamiliar to (most of) the participants. Familiarity with the stimulus was further checked in a postquestionnaire, with 87.88% stating to not know the track at all, 6.06% reporting to have possibly recognized the track, 0% stating to know the track, and 6.06% to being indecisive. Taking the above‐described criteria into account, the song *International Dateline* by *Ladytron* (2005), with an original tempo of 168 BPM, was selected. As the duration of the track did not cover the complete length of a condition (i.e., 4 min), the chorus part in the middle of the song was copied and repeated at the end of the track (using Audacity software, see http://audacity.sourceforge.net) when it had to undergo substantial tempo increases (up to 200 BPM). Beats were automatically detected using BeatRoot^38^ and manually checked afterward.

### Apparatus

Participants were equipped with two iPods (4th generation); one attached to each ankle. Using the Sensor Monitor Pro application on the iPods, data from the iPod accelerometers and gyroscopes were streamed wirelessly at 100 Hz to a 7′′ tablet (Panasonic Roughpad FZ‐M1) running Windows 8.1. The tablet was strapped to a backpack, together with a sonar (MaxBotix LV‐MaxSonar‐EZ: MB1010) pointing to the right of the runner and connected to the tablet through a Teensy 3.1 microcontroller. Twenty‐nine 1.9‐m vertical marker rods were placed on the right side of the running track (289 m) with a spacing of 9.97 meters. The sonar and the rods were used to calculate the runners’ speed in a postprocessing phase.

The wireless connection between the tablet and iPods was provided through a Wi‐Fi router (TP‐Link M5360), firmly strapped to the backpack, ensuring reliable communication between the iPods and the tablet. On the tablet, Max/MSP from Cycling74’ was running together with a patch specifically designed to read out the sensor data, implement the different conditions, and store the data. The audio output was provided through Sennheiser HD 215 headphones. Music tempo was manipulated using the MAX/MSP elastic∼ object by Simon Adcock, which allows for tempo alterations of ±/–100% of the original music tempo without pitch modifications.

### Procedure

The experiments took place at an indoor track‐and‐field site (Flanders Sports Arena, Ghent, Belgium). Participants were equipped with the iPods, headphones, and a backpack containing the tablet, sonar, and Wi‐Fi router. They were asked to run four times for 4 minutes. No information was distributed concerning the real purpose of the experiment and all participants ran solo. After each 4‐min running session, a break of at least 5 min was introduced to enable them to recover sufficiently. During the break, the participant was asked to take sufficient rest. After he/she expressed feeling approximately as fit as at the start of the experiment, the participant initiated the following running session. Between sessions, participants were asked to fill out the Borg Rating of Perceived Exertion (RPE) Scale^39^, ^40^ and indicate how heavy the effort had been during the exercise, ranging from 6 (“no exertion at all”) to 20 (“maximal exertion”). In addition, they rated the level of physical enjoyment of the previously performed exercise on the 8‐item version of the Physical Activity Enjoyment Scale (PACES),^41^, ^42^ a single‐factor 7‐point Likert scale to assess the level of enjoyment during physical activity in adults across exercise modalities.

In the training session, participants were asked to run at their self‐paced cadence without musical accompaniment. This session was included to warm up and get acquainted with the running track and was not taken into account in the analysis. In the first running session (*no music* condition), no music was played and participants were asked to run at their self‐paced cadence. Next, a familiarization task took place where the participants first listened to the music track without moving to it. In the second session (*uninstructed* condition), participants exercised at their self‐paced cadence again, this time accompanied by music with a tempo matching their cadence assessed during the last 120 steps taken in the previous condition.^a^ During the third session (*instructed* condition), the same stimulus was presented, yet participants were instructed to “match their running cadence with the tempo of the music.” As exercise behavior might be influenced by a foreseen completion of the task (e.g., speeding up near the end of the experiment), a fourth and final condition was added, in which participants were asked to run at their self‐pace cadence once more with musical accompaniment. This condition was merely implemented to control for confounding effects of anticipated task completion and was not taken into account in the analysis.

At the end of the experiment, participants filled out a questionnaire on personal background, music education, and sports training. In addition, participants’ perception of their personal level of movement‐to‐music matching behavior in the instructed condition was assessed, as well as to what extent they generally tend to match their cadence to musical tempi outside the experimental setting.

### Data analysis

To test the effect of the specific condition on running behavior, the following features were calculated: cadence, speed, step length, tempo entrainment, mean relative phase angle (rPA), and resultant vector length (RVL). Before the calculation of all features, the initial 60 s of each run was discarded to avoid a start‐up effect. The final 30 s of each run was ignored as well, to eliminate altered running behavior due to the anticipated ending (e.g., slowing down or speeding up). Movement features were calculated as follows.

#### Cadence (SPM)

Running cadence was calculated in real time using the acceleration data acquired by the iPods. A change in the movement direction of the leg, detected by the gyroscope, was identified as a step. The tempo intervals of eight consecutive steps of the same leg were used to calculate cadence (SPM) in a moving average manner.

#### Step length (m)

Step length was calculated in real time as the distance measured from the heel print of one foot to the heel print of the other foot.

#### Speed (km/h)

The distance measurements provided by the sonar were used in a postprocessing phase to evaluate running speed. When the runner passed along the rods, placed on the right side of the track, a distance minimum was detected. Through computation of the time between the minima, that is, between the rods, average speed was determined. The analog signal was sampled at 250 Hz and digitized using the Teensy microcontroller.

#### Tempo entrainment (%)

Another measure consisted of the percentage of tempo‐entrained steps during the conditions with music. A step taken in a tempo sufficiently close to the music tempo (maximum of 1% difference between SPM and BPM) at that specific moment is regarded as a tempo‐entrained step. The tempo entrainment score is the percentage of tempo‐entrained steps out of the total number of steps.

#### Mean rPA (degrees)

The mean rPA is a measure of the timing of the footfall relative to the closest beat and can be expressed as either a positive (footfall after the beat) or a negative (footfall before the beat) angle in degrees. The rPA of 0° refers to a footfall that is exactly timed on the beat, and an angle of 180° refers to a footfall that is timed precisely in between two beats. Such an rPA can be calculated for each step with the following equation (*St* refers to the time of a step, *B1* refers to the time of the beat before the step, and *B2* refers to the time of the beat after the step)

$$\;\phi =360*\frac{St-B1}{B2-B1},$$

after which the circular mean of all rPAs can be calculated.^43^ The mean of all rPAs is only of interest if there is a sufficient amount of consistency in entrainment, which is expressed by the RVL (see below). Therefore, only mean rPA values that correspond to RVL values of ≥0.75 are considered in the analysis.

#### RVL (value from 0 to 1)

The RVL expresses the coherence or stability of the rPA over time.^44^ If the distribution of the rPA over time is narrow (when all phase angles are clustered around the mean), it leads to a high RVL (maximum value 1), which indicates highly consistent entrainment. In the case of a broad or multimodal rPA over time, RVL is low (minimum value toward 0), indicating no auditory–motor coupling or entrainment with the music. Additionally, participants were divided into entrainers and nonentrainers using a cutoff of ≥0.75, based on previous research (e.g., see Refs. 29 and 37).

For all movement features, a 3 × 2 mixed‐design ANOVA with *condition* as within‐subjects factor (no music, uninstructed, and instructed) and *sex* as a between‐subject factor was performed. The no music condition could not be taken into account for features depending on musical parameters (e.g., tempo entrainment, rPA, and RVL). An independent samples *t*‐test was performed to check for the effects of musical training on tempo entrainment and RVL, while one‐way ANOVA was executed to check for differences between participants reporting to habitually run with, without, or both with and without music. Friedman's ANOVA was employed to check for differences in PACES and BORG RPE‐scale ratings between conditions and one‐way ANOVA was used to examine the potential effects of perceived alignment on tempo entrainment.

## Results

### Cadence

A significant main effect of condition was revealed, *F*(2,62) = 18.12, *P* < 0.001. Contrasts showed that running cadence was significantly lower in the no music condition (*M* = 168.78; SE = 1.53) compared with the other conditions: uninstructed (*M* = 170.89; SE = 1.54), *F*(1,31) = 42.61, *P* < 0.001, *η* ^2^ = 0.58, and instructed (*M* = 170.30; SE = 1.45), *F*(1,31) = 13.07, *P* = 0.001, *η* ^2^ = 0.30. No significant difference was found between the instructed and uninstructed conditions, *F*(1,31) = 3.31, *P* = 0.08, *η* ^2^ = 0.10.

A significant main effect of sex was obtained as well, revealing higher cadence rates for females (*M* = 173.34; SE = 1.86) than males (*M* = 165.97; SE = 2.03), *F*(1,31) = 7.18, *P* = 0.009, *η* ^2^ = 0.18.

**Figure 1:**
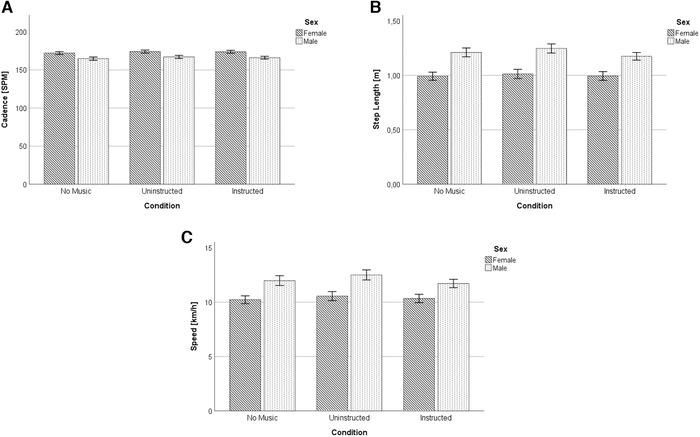
Results on cadence (A), step length (B), and speed (C) data of male and female runners, for the no music, uninstructed, and instructed conditions. Data presented are the mean ± SE.

There was no significant effect of the condition × sex interaction, *F*(2,62) = 0.31, *P* = 0.73 (Fig. 1A).

### Step length

We obtained a significant main effect of condition, *F*(2,62) = 13.43, *P* < 0.001, demonstrating larger step lengths in the uninstructed condition (*M* = 1.12; SE = 0.04) compared with the no music (*M* = 1.09; SE = 0.03), *F*(1,31) = 18.41, *P <* 0.001, *η* ^2^ = 0.37, and the instructed one (*M* = 1.08; SE = 0.03), *F*(1,31) = 21.25, *P <* 0.001, *η* ^2^ = 0.41. No significant difference was obtained between the no music and instructed conditions, *F*(1,31) = 3.01, *P* = 0.09, *η* ^2^ = 0.09.

There was a significant main effect of sex, revealing larger step lengths for males (*M* = 1.21; SE = 0.04) compared with their female counterparts (*M* = 1.00; SE = 0.04), *F*(1,31) = 14.39, *P* = 0.001, *η* ^2^ = 0.31.

In addition, a significant effect of the condition × sex interaction was obtained, indicating that males make larger differences in step length between the uninstructed and instructed condition compared with their female counterparts, *F*(2,62) = 4.88, *P* = 0.01 (Fig. 1B).

### Speed

There was a significant main effect of condition, *F*(2,62) = 15.21, *P* < 0.001, demonstrating faster running behavior in the uninstructed condition (*M* = 11.44; SE = 0.35) compared with the no music (*M* = 11.02; SE = 0.32), *F*(1,31) = 40.40, *P* < 0.001, *η* ^2^ = 0.56, and the instructed (*M* = 10.96; SE = 0.29), *F*(1,31) = 20.86, *P* < 0.001, *η* ^2^ *= *0.41, conditions. No significant difference was found between the no music and instructed conditions, *F*(1,31) = 0.43, *P* = 0.52, *η* ^2^ = 0.01.

There was a significant main effect of sex, revealing higher speed levels for males (*M* = 12.07; SE = 0.42) compared with their female counterparts (*M* = 10.37; SE = 0.38), *F*(1,31) = 8.87, *P* = 0.006, *η* ^2^ = 0.22.

A significant effect of the condition × sex interaction was obtained as well, showing that larger speed differences between the uninstructed and instructed conditions were made by males than by females, *F*(2,62) = 4.36, *P* = 0.02 (Fig. 1C).

### Tempo entrainment

Tempo entrainment proved to be significantly higher in the instructed (*M* = 0.62; SD = 0.28) compared with the uninstructed condition (*M* = 0.51; SD = 0.30), *F*(1,31) = 7.68, *P* = 0.009, *η* ^2^ *= *0.20.

**Figure 2:**
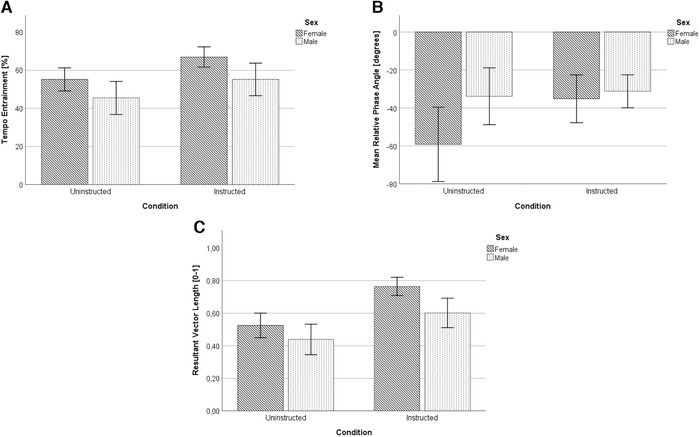
Results on tempo entrainment (A), mean relative phase angle (B), and resultant vector length (C) data of male and female runners, for both the uninstructed and instructed conditions. Data presented are the mean ± SE.

There was no significant main effect of sex, *F*(1,31) = 1.36, *P* = 0.25, *η* ^2^ = 0.04, nor was there a significant effect of the condition × sex interaction, *F*(1,31) = 0.07, *P* = 0.79, *η* ^2^ = 0.003 (Fig. 2A).

### Mean rPA (rPA)

A significant difference was found for mean rPA, indicating footfalls more closely match musical beats in the instructed condition (*M* = −33.75; SD = 36.63) compared with the uninstructed one (*M* = −47.69; SD = 42.16), *F*(1,7) = 7.32, *P* = 0.03, *η* ^2^ = 0.52.

No significant main effect of sex, *F*(1,31) = 0.14, *P* = 0.72, *η* ^2^ = 0.02, or of the condition × sex interaction, *F*(1,31) = 0.03, *P* = 0.87, *η* ^2^ = 0.004, was obtained. (Fig. 2B).

### RVL

The RVL was shown to be significantly higher in the instructed (*M = *0.69; SD = 0.30) compared with the uninstructed condition (*M* = 0.49; SD = 0.34), *F*(1,31) = 17.18, *P* < 0.001, *η* ^2^ = 0.13.

No significant main effect of sex, *F*(1,31) = 1.55, *P* = 0.22, *η* ^2^ = 0.05, or of the condition × sex interaction, *F*(1,31) = 0.63, *P* = 0.44, *η* ^2^ = 0.0004, was found (Fig. 2C).

### Musical experience

Significantly higher levels of tempo entrainment and RVL were obtained for musically trained participants (tempo entrainment: *M* = 0.68, SD = 0.16; and RVL: *M* = 0.73, SD = 0.20), compared with their untrained counterparts (tempo entrainment: *M* = 0.49, SD = 0.29, *t*(31) = −2.12, *P* = 0.04, *η* ^2^ *= *0.13; and RVL: *M* = 0.51, SD = 0.30, *t*(31) = −2.33, *P* = 0.03, *η* ^2^ *= *0.15).

No significant effects for either of these parameters (tempo entrainment, *F*(2,30) = 0.17, *P* = 0.85; and RVL, *F*(2,30) = 0.002, *P* = 0.99) were found between participants who reported habitually running with or without music, or those who indicated both running with and without musical accompaniment.

### PACES

A significant main effect of the ratings was obtained, *χ* ^2^(2) = 15.53, *P <* 0.001, and Wilcoxon tests were used to follow up on this finding. After Bonferroni correction, it appeared that ratings for the no music condition (*Mdn* = 4.63) were significantly lower compared with the other two conditions: uninstructed, (*Mdn* = 5.00), *z* = −3.77, *P* < 0.001, *η* ^2^ *= *0.23; and instructed, (*Mdn* = 5.00), *z* = −2.61, *P* = 0.009, *η* ^2^ *= *0.10. No significant differences were found between the uninstructed and instructed conditions, *z* = −0.97, *P* = 0.33, *η* ^2^ *= *0.01.

### Borg RPE

No significant change in Borg RPE‐scale ratings over the conditions was obtained, *χ* ^2^(2) = 0.29, *P* = 0.87.

### Perception of alignment

Of all participants, none of them answered negatively when asked if they believed they had aligned their running movements with the music in the *instructed* condition; 15.15% replied that they did not know whether they did so or not; 21.21% answered that, at times, they indeed aligned with the music; and 63.64% reported to have aligned their running cadence with the music tempo most of the time. We checked for differences in auditory–motor coupling between these three groups of participants. However, no significant effect of perceived alignment was found for tempo entrainment, *F*(2,30) = 1.10, *P* = 0.35, rPA, *F*(2,16) = 0.24, *P* = 0.79, or RVL, *F*(2,30) = 1.12, *P* = 0.34.

When asked about their entrainment behavior in daily life, 39.39% of all participants did not know whether they entrain their running behavior to the perceived music or generally do not run to music; 3.03% of them reported not entraining to music in daily life; 45.45% disclosed to occasionally entraining with the music; and 12.12% pointed out generally entraining to music while running.

## Discussion

In this study, we aimed to investigate the difference between instructed and uninstructed (or spontaneous) entrainment of running to music. Our findings showed that instruction resulted in a significant increase in the level of movement‐to‐music entrainment when compared with the same running task without instruction. When the runners were not instructed to align their movements with the musical beats, only 33.33% of the participants spontaneously entrained to the stimulus. Yet, when instructions to “match running cadence with musical tempo” were given, 57.58% of the runners entrained with the beats. Such results are rather surprising, since in walking research usually larger population ratios display spontaneous entrainment to musical stimuli in tempi close to preferred exercising paces (e.g., 40%, see Ref. 33; about 50%, see Ref. 37; and about 60%, see Ref. 29). Moreover, studies on walking also revealed higher levels of entrainment with instruction (e.g., 74%, see Ref. 33; and up to 93%, see Ref. 29). However, running is a more strenuous effort and involves different biomechanics; although it is a natural extension of walking, running involves increased velocities, joint range of motion, forces, muscle activity, joint moments, and joint powers as compared with walking. Thus, running stresses the mechanics of the body to a greater extent, as such also increasing the risk of related injury.^45^

Even though, on average, entrainment was lower compared with previous walking research, a similar difference between spontaneous and instructed entrainment was demonstrated for runners, with higher levels of tempo entrainment as well as RVL (an alternative measure of entrainment) when instructed to match exercise behavior to musical beats. In addition, despite the fact that footfall instances generally preceded musical beats, rPAs decreased with instruction, indicating a closer match to beat occurrences. However, even when instructions to match running behavior to music were given, entrainment frequency remained rather low, a finding similar to previous work showing low movement‐to‐music coupling frequencies after participants were instructed to adapt movements of the entire body to the music.^46^ These results support the hypothesis that matching movements to musical beats may not be a simple, low‐level task; entrainment in itself may be cognitively demanding,^46^ most particularly for individuals who have difficulty perceiving the beat in music.^33^, ^47^, ^48^, ^49^

Besides entrainment, also cadence, step length, and speed—three key performance measures of running—were scrutinized. All three features proved to increase in the uninstructed condition when compared with the condition without musical accompaniment. This is in line with the idea that music is capable of increasing exercise intensity and endurance.^1^, ^2^, ^3^, ^4^, ^5^ Although the precise mechanisms through which music can boost performance still require further investigation, this effect might be (partly) explained by the propensity of music to heighten arousal.^34^, ^50^, ^51^ In the instructed condition, a similar increase in cadence occurred. However, compared with the silent condition, step length and speed did not significantly change when instructed to run to the beat. These results are consistent with previous results on walking behavior, demonstrating that instructing participants to move to the beat elicited slower and shorter strides than when instruction was absent.^33^ They are also in accordance with earlier findings indicating that, when compared with stride‐based pacing, step‐based pacing leads to more stable auditory–motor coordination in both walking and running.^52^ Consequently, although a number of studies demonstrated that auditory–motor coupling improved performance in motor tasks,^11^, ^12^, ^18^ our findings suggest that entrainment as such does not necessarily speed up recreational runners or lengthen their steps, as this seems to depend on the presence/absence of instruction. The fact that instructed entrainment did not lead to an increase in speed and step length, whereas spontaneous entrainment did, might be related to the idea that instruction results in more goal‐directed behavior, as such directing the focus to the achievement of entrainment and suppressing possible arousal effects caused by the auditory accompaniment. The combination of our results on entrainment as well as cadence, step length, and speed indeed corresponds with cognitive motor learning models, suggesting that explicit instruction in motor control contexts may lead to more intentional behavior and promote greater deliberate control of movement compared with baseline and, in turn, disrupt movement in line with the conscious processing hypothesis.^33^, ^53^ This hypothesis is applied to healthy populations, such as the recreational runners studied here. However, it might not hold for specific gait‐disordered populations, since previous clinical work indicated that rhythmic auditory cues can, for instance, help Parkinson's disease patients to take faster and longer (as well as less variable) strides, even when instructed to entrain.^54^

Although rPAs decreased with instruction (implying a closer match of footsteps and beat instances), negative angles were exhibited both with and without instruction to entrain to the music. As a negative rPA implies footfalls to occur before the beat, a prediction error minimization process occurred; runners presumably relied on anticipatory mechanisms, which allowed them to predict the beats and coordinate their own anticipated actions with these predictions.^55^ This idea is supported by previous research revealing positive correlations between prediction/tracking ratios and the acuity of auditory imagery for timing.^56^ It has been suggested that the formation of auditory images largely relies on working memory.^57^, ^58^, ^59^ Moreover, activation of the corresponding brain areas was observed during auditory imagery.^60^

Although music is believed to distract from feelings of fatigue and discomfort,^6^, ^7^ self‐rated perceived physical fatigue did not change over conditions. This is possibly the result of the short duration of the running tasks, in combination with the low‐to‐moderate intensity of the exercise, as such not prompting significant feelings of fatigue or exhaustion. Yet, levels of physical enjoyment did improve in the presence of a musical stimulus, which is in accordance with the general idea that music can alter mood states and stimulate motivation.^7^, ^8^ As corporeal coupling to musical stimuli can support the feeling of agency,^61^ further igniting motivational components,^29^ we did expect to obtain increased levels of physical enjoyment in the instructed condition compared with the uninstructed one. Yet, no such effects were found, suggesting that instruction as such did not influence runners’ enjoyment of the exercise.

Since some previous research provided (direct or indirect) proof to indicate that women are more responsive to musical stimuli than men,^8^, ^28^, ^62^, ^63^ runners’ sex was taken into account in the analysis. In contrast with such evidence, our results did not reveal differences between men and women regarding entrainment behavior. However, larger differences in speed and step length between the uninstructed and instructed conditions were exhibited for male runners, possibly indicating that they were more responsive to the instruction. Yet, this is a matter of some speculation and other factors might have been at play as well. On average, women were, for instance, shown to exercise more often to music than men, as well as to prefer other music styles,^64^ and experience different affects and levels of motivation while doing so.^8^, ^63^

An effect of musical training was retrieved, demonstrating higher levels of tempo entrainment and increased RVLs for musically trained runners compared with their untrained counterparts. As such, musical experience might be suggested to facilitate auditory–motor coupling, which is in consonance with previous finger‐tapping research indicating greater synchronization accuracy for musicians than nonmusicians; musicians synchronized more flexibly while tapping, while nonmusicians showed greater temporal rigidity.^65^ The observed decreased ability of nonmusically trained individuals to entrain to musical beats might result from weaker auditory–motor integration.^49^, ^66^ Findings in cognitive psychology also suggested that successful adaptation to stimuli is mediated by the level of regularity in the specific environment (i.e., making it more predictable) and the opportunity to have obtained sufficient practice in such a setting.^67^ This would thus imply that individuals who obtained more musical practice would adapt more efficiently to a regular (thus predictable) beat, which was indeed confirmed by our results.

In our current study, the type of entrainment (or synchronization) refers to the period matching of two (or more) dynamical systems. Although most research on the alignment of running and walking behavior and musical beats focused on period matching, we could also have opted to target phase‐locking (footfall instances occurring in phase with the musical beats). However, as research indicated that footfall instances of running and walking behavior usually occur before or after the beat of the music (e.g., see Refs. 28, 29, and 37), tempo entrainment (or tempo synchronization) was studied here.

A within‐subjects design was selected to control for a wide range of features previously indicated to possibly impact auditory–motor coupling, such as biomechanical characteristics of the individual subjects,^45^ preferred running pace,^19^ age,^8^ training level,^34^, ^35^ and music preference.^1^, ^64^ As a result, the order of the conditions could not be counterbalanced; however, measures were taken to circumvent potential associated effects. To prevent confounding effects of exhaustion and fatigue, only runners who reported to be fit to run comfortably for at least 30 min without feeling exhausted were invited. In addition, participants were asked to take sufficient rest (and were required to pause for at least 5 min) between running tasks. Moreover, reported fatigue was analyzed, demonstrating no differences between conditions. We also aimed to control for possible effects of familiarity with the musical stimulus through the inclusion of a familiarization task before the first running session with musical accompaniment.

It should be stressed that this study focused on recreational runners running at a self‐paced tempo. However, as less‐trained exercisers were shown to depend to a greater extent on the positive feeling states generated by music, while trained exercisers generally tend to focus on the tasks and specifics of their training,^34^, ^35^ current findings might not be applicable to more professionally trained runners. Moreover, when studying higher levels of running intensity, different results might be obtained. When high workloads are undertaken, the exerciser's attention could be shifted toward the painful or fatiguing effects of the exercise,^20^, ^21^, ^22^, ^23^, ^24^ which might result in lower levels of entrainment with the musical beats.

Overall, this study demonstrates the impact of instruction on running performance. Compared with a similar running task without instruction, results showed higher levels of tempo entrainment, lower speeds, and shorter step lengths of recreational runners when instructed to match exercise with musical tempo. Our results are especially relevant to recreational runners, as their performance might be mediated through intentionality. We would, however, expect that instruction might not impact a runner's entrainment basin. Previously, recreational runners were shown to spontaneously adapt their running cadence up to 2% of their baseline cadence to tempo changes in music.^28^ As instruction did not seem to impact running cadence in the current study, we would expect a similar entrainment basin both with and without instructions to adapt to the musical beats. However, this is a matter of some speculation and would benefit from further study. Finally, our findings might prove to be interesting to trainers and researchers as well, since the desired exercise output might, at least to a certain extent, depend on what exercisers/participants were exactly asked to do. As larger step lengths can negatively impact loading of the lower extremity joints,^68^, ^69^, ^70^ instruction might also prove its value in the light of prevention and treatment of common running‐related injuries.

## Author contributions

E.V.D., J.B., and V.L. participated in research design and conducted experiments. E.V.D. and J.B. performed data analysis. E.V.D. wrote the first draft. J.B. and V.L. edited or contributed to the writing of the manuscript.

## Competing interests

The authors declare no competing interests.

## Footnotes

a The final 40 steps were excluded in order to disregard possible alterations in cadence due to the anticipation of the end of the condition.

## References

[1] Priest, D.L. & C.I. Karageorghis . 2008. A qualitative investigation into the characteristics and effects of music accompanying exercise. Eur. Phy. Educ. Rev. 14: 347–366.

[2] Edworthy, J. & H. Waring . 2006. The effects of music tempo and loudness level on treadmill exercise. Ergonomics 49: 1597–1610. 17090506 10.1080/00140130600899104

[3] Rendi, M. , A. Szabo & T. Szabó . 2008. Performance enhancement with music in rowing sprint. Sport Psychol. 22: 175–182.

[4] Atkinson, G. , D. Wilson & M. Eubank . 2004. Effects of music on work‐rate distribution during a cycling time trial. Int. J. Sports Med. 25: 611–615. 15532005 10.1055/s-2004-815715

[5] Maddigan, M.E. , K.M. Sullivan , I. Halperin , *et al*. 2019. High tempo music prolongs high intensity exercise. PeerJ 8: e6164. 10.7717/peerj.6164 PMC6329333 30643679

[6] Yamashita, S. , K. Twai , T. Aktmoto , *et al*. 2006. Effects of music during exercise on RPE, heart rate and the autonomic nervous system. J. Sports Med. Phys. Fitness 46: 425–430. 16998447

[7] Shaulov, N. & D. Lufi . 2009. Music and light during indoor cycling. Percept. Mot. Skills 108: 597–607. 19544965 10.2466/PMS.108.2.597-607

[8] Priest, D.L. , C.I. Karageorghis & N.C.C. Sharp . 2004. The characteristics and effects of motivational music in exercise settings: the possible influence of gender, age, frequency of attendance, and time of attendance. J. Sports Med. Phys. Fitness 44: 77–86. 15181394

[9] Lim, H.B.T. , C.I. Karageorghis , L.M. Romer , *et al*. 2014. Psychophysiological effects of synchronous versus asynchronous music during cycling. Med. Sci. Sports Exerc. 46: 407–413. 24441216 10.1249/MSS.0b013e3182a6378c

[10] Hsu, D.Y. , L. Huang , L.F. Nordgren , *et al*. 2015. The music of power: perceptual and behavioral consequences of powerful music. Soc. Psychol. Personal Sci. 6: 75–83.

[11] Terry, P.C. , C.I. Karageorghis , A. Mecozzi Saha , *et al*. 2012. Effects of synchronous music on treadmill running among elite triathletes. J. Sci. Med. Sport 15: 52–57. 21803652 10.1016/j.jsams.2011.06.003

[12] Karageorghis, C.I. , D. Mouzourides , D.L. Priest , *et al*. 2009. Psychophysical and ergogenic effects of synchronous music during treadmill walking. J. Sport Exerc. Psychol. 31: 18–36. 19325186 10.1123/jsep.31.1.18

[13] Kenyon, G.P. & M.H. Thaut . 2003. Rhythm‐driven optimization of motor control. Recent Res. Dev. Biomech. 1: 29–47.

[14] Smoll, F.L. & R.W. Schultz . 1982. Accuracy of motor behavior in response to preferred and nonpreferred tempos. J. Hum. Mov. Stud. 8: 123–138.

[15] Rossignol, S. & G. Melvill‐Jones . 1976. Audiospinal influences in man studied by the H‐reflex and its possible role in rhythmic movement synchronized to sound. Electroencephalogr. Clin. Neurophysiol. 41: 83–92. 58771 10.1016/0013-4694(76)90217-0

[16] Bood, R.J. , M. Nijssen , J. van der Kamp , *et al*. 2013. The power of auditory–motor synchronization in sports: enhancing running performance by coupling cadence with the right beats. PLoS One 8: e70758. 23951000 10.1371/journal.pone.0070758 PMC3737354

[17] Bacon, C.J. , T.R. Myers & C.I. Karageorghis . 2012. Effect of music‐movement synchrony on exercise oxygen consumption. J. Sports Med. Phys. Fitness 52: 359–365. 22828457

[18] Simpson, S.D. & C.I. Karageorghis . 2006. The effects of synchronous music on 400‐m sprint performance. J. Sports Sci. 24: 1095–1102. 17115524 10.1080/02640410500432789

[19] Karageorghis, C.I. & D.L. Priest . 2012. Music in the exercise domain: a review and synthesis (Part II). Int. Rev. Sport Exerc. Psychol. 5: 44–66. 22577472 10.1080/1750984X.2011.631026 PMC3339578

[20] Rejeski, W.J. 1985. Perceived exertion: an active or passive process? J. Sport Exerc. Psychol. 7: 371–378.

[21] Nethery, V.M. 2002. Competition between internal and external sources of information during exercise: influence on RPE and the impact of the exercise load. J. Sports Med. Phys. Fitness 42: 172–178. 12032412

[22] Tenenbaum, G. 2005. The study of perceived and sustained effort: concepts, research findings, and future directions. In Handbook of Research on Applied Sport Psychology. D. Hackfort , J. Duda & R. Lidor , Eds.: 335–349. Morgantown: Fitness Information Technology.

[23] Razon, S. , I. Basevitch , W. Land , *et al*. 2009. Perception of exertion and attention allocation as a function of visual and auditory conditions. Psychol. Sport Exerc. 10: 636–643.

[24] Hutchinson, J.C. & G. Tenenbaum . 2007. Attention focus during physical effort: the mediating role of task intensity. Psychol. Sport Exerc. 8: 233–245.

[25] Styns, F. , L. van Noorden , D. Moelants , *et al*. 2007. Walking on music. Hum. Mov. Sci. 26: 769–785. 17910985 10.1016/j.humov.2007.07.007

[26] Mendonça, C. , M. Oliveira , L. Fontes , *et al*. 2014. The effect of instruction to synchronize over step frequency while walking with auditory cues on a treadmill. Hum. Mov. Sci. 33: 33–42. 24576706 10.1016/j.humov.2013.11.006

[27] Large, E.W. 2000. On synchronizing movements to music. Hum. Mov. Sci. 19: 527–566.

[28] Van Dyck, E. , B. Moens , J. Buhmann , *et al*. 2015. Spontaneous entrainment of running cadence to music tempo. Sports Med. Open 1: 15. 26258007 10.1186/s40798-015-0025-9 PMC4526248

[29] Moumdjian, L. , B. Moens , E. Vanzeir , *et al*. 2019. A model of different cognitive processes during spontaneous and intentional coupling to music in multiple sclerosis. Ann. N.Y. Acad. Sci. 1445: 27–38. 30865313 10.1111/nyas.14023

[30] Lopresti‐Goodman, S.M. , M.J. Richardson , P.L. Silva , *et al*. 2008. Period basin of entrainment for unintentional visual coordination. J. Mot. Behav. 40: 3–10. 18316292 10.3200/JMBR.40.1.3-10

[31] Schmidt, R.C. & M.J. Richardson . 2008. Dynamics of Interpersonal Coordination. Berlin: Springer‐Verlag.

[32] Strogatz, S.H. 1994. Nonlinear Dynamic and Chaos: with Applications to Physics, Biology, Chemistry, and Engineering. Cambridge: Perseus Books.

[33] Leow, L.A. , K. Waclawik & J.A. Grahn . 2018. The role of attention and intention in synchronization to music: effects on gait. Exp. Brain Res. 236: 99–115. 29075835 10.1007/s00221-017-5110-5

[34] Brownley, K.A. , R.G. McMurray & A.C. Hackney . 1995. Effects of music on physiological and affective responses to graded treadmill exercise in trained and untrained runner. Int. J. Psychophysiol. 19: 193–201. 7558986 10.1016/0167-8760(95)00007-f

[35] Mohammadzadeh, H. , B. Tartibiyan & A. Ahmadi . 2008. The effects of music on the perceived exertion rate and performance of trained and untrained individuals during progressive exercise. Facta Univ. Ser. Phys. Educ. Sport 6: 67–74.

[36] Faul, F. , E. Erdfelder , A.‐G. Lang , *et al*. 2007. G* power 3: a flexible statistical power analysis program for the social, behavioral, and biomedical sciences. Behav. Res. Methods 39: 175–191. 17695343 10.3758/bf03193146

[37] Buhmann, J. , F. Desmet , B. Moens , *et al*. 2016. Spontaneous velocity effect of musical expression on self‐paced walking. PLoS One 11: e0154414. 27167064 10.1371/journal.pone.0154414 PMC4864300

[38] Dixon, S. 2007. Evaluation of the audio beat tracking system BeatRoot. J. New Music Res. 36: 39–50.

[39] Ajzen, I. & M. Fishbein . 1977. Attitude–behavior relations: a theoretical analysis and review of empirical research. Psychol. Bull. 84: 888–918.

[40] Borg, G. 1998. Borg's Perceived Exertion and Pain Scales. Champaign, IL: Human Kinetics.

[41] Kendzierski, D. & K.J. DeCarlo . 1991. Physical activity enjoyment scale: two validation studies. J. Sport Exerc. Psychol. 13: 50–64.

[42] Mullen, S.P. , E.A. Olson , S.M. Phillips , *et al*. 2011. Measuring enjoyment of physical activity in older adults: invariance of the Physical Activity Enjoyment Scale (PACES) across groups and time. Int. J. Behav. Nutr. Phys. Act. 8: 1–9. 21951520 10.1186/1479-5868-8-103 PMC3206413

[43] Berens, P. 2009. CircStat: a MATLAB toolbox for circular statistics. J. Stat. Softw. 31: 10.

[44] Mormann, F. , K. Lehnertz , P. David , *et al*. 2000. Mean phase coherence as a measure for phase synchronization and its application to the EEG of epilepsy patients. Physica D 144: 358–369.

[45] Ounpuu, S. 1994. The biomechanics of walking and running. Clin. Sports Med. 13: 843–863. 7805110

[46] Burger, B. , M.R. Thompson , G. Luck , *et al*. 2014. Hunting for the beat in the body: on period and phase locking in music‐induced movement. Front. Hum. Neurosci. 8: 903. 25426051 10.3389/fnhum.2014.00903 PMC4224089

[47] Fujii, S. & G. Schlaug . 2013. The Harvard Beat Assessment Test (H‐BAT): a battery for assessing beat perception and production and their dissociation. Front. Hum. Neurosci. 7: 771. 24324421 10.3389/fnhum.2013.00771 PMC3840802

[48] Launay, J. , M. Grube & L. Stewart . 2014. Dysrhythmia: a specific congenital rhythm perception deficit. Front. Psychol. 5: 18. 24550854 10.3389/fpsyg.2014.00018 PMC3913998

[49] Sowinski, J. & S. Dalla Bella . 2013. Poor synchronization to the beat may result from deficient auditory‐motor mapping. Neuropsychologia 51: 1952–1963. 23838002 10.1016/j.neuropsychologia.2013.06.027

[50] Becker, N. , S. Brett , C. Chambliss , *et al*. 1994. Mellow and frenetic antecedent music during athletic performance of children, adults, and seniors. Percept. Mot. Skills 79: 1043–1046. 7870490 10.2466/pms.1994.79.2.1043

[51] Karageorghis, C.I. , K. Drew & P. Terry . 1996. Effects of pretest stimulative and sedative music on grip strength. Percept. Mot. Skills 83: 1347–1352. 9017751 10.2466/pms.1996.83.3f.1347

[52] Nijs, A. , M. Roerdink & P.J. Beek . 2020. Cadence modulation in walking and running: pacing steps or strides? Brain Sci. 10: 273. 10.3390/brainsci10050273 PMC7288070 32370091

[53] Masters, R.S.W. 1992. Knowledge, knerves, and know‐how: the role of explicit versus implicit knowledge in the breakdown of a complex motor skill under pressure. Br. J. Psychol. 83: 343–358.

[54] Hausdorff, J.M. 2007. Gait dynamics, fractals and falls: finding meaning in the stride‐to‐stride fluctuations of human walking. Hum. Mov. Sci. 26: 555–589. 17618701 10.1016/j.humov.2007.05.003 PMC2267927

[55] Keller, P.E. 2008. Joint action in music performance. In Enacting Intersubjectivity: A Cognitive and Social Perspective to the Study of Interactions. F. Morganti , A. Carassa & G. Riva , Eds.: 205–221. Amsterdam: IOS Press.

[56] Pecenka, N. & P.E. Keller . 2009. The relationship between auditory imagery and musical synchronization abilities in musicians. In Proceedings of the 7th Triennial Conference of European Society for the Cognitive Sciences of Music. J. Louhivuori , T. Eerola , S. Saarikallio *et al*., Eds.:409–414. Jyväskylä: University of Jyväskylä.

[57] Baddeley, A. & R. Logie . 1992. Auditory imagery and working memory. In Auditory Imagery. D. Reisberg , Ed.: 179–197. Hillsdale: Erlbaum.

[58] Smith, J. , M. Wilson & D. Reisberg . 1995. The role of subvocalization in auditory imagery. Neuropsychologia 33: 1433–1454. 8584179 10.1016/0028-3932(95)00074-d

[59] Hubbard, T.L. 2010. Auditory imagery: empirical findings. Psychol. Bull. 136: 302–329. 20192565 10.1037/a0018436

[60] Aleman, A. & M. van't Wout . 2004. Subvocalization in auditory–verbal imagery: just a form of motor imagery? Cogn. Process. 5: 228–231.

[61] Fritz, T.H. , S. Hardikar , M. Demoucron , *et al*. 2013. Musical agency reduces perceived exertion during strenuous physical performance. Proc. Natl. Acad. Sci. USA 110: 17784–17789. 24127588 10.1073/pnas.1217252110 PMC3816438

[62] Cole, Z. & H. Maeda . 2015. Effects of listening to preferential music on sex differences in endurance running performance. Percept. Mot. Skills 121: 390–398. 26447745 10.2466/06.PMS.121c20x9

[63] Karageorghis, C.I. , D.L. Priest , L.S. Williams , *et al*. 2010. Ergogenic and psychological effects of synchronous music during circuit‐type exercise. Psychol. Sport Exerc. 11: 551–559.

[64] Hallett, R. & A. Lamont . 2016. Music use in exercise: a questionnaire study. Media Psychol. 20: 658–684.

[65] Scheurich, R. , A. Zamm & C. Palmer . 2018. Tapping into rate flexibility: musical training facilitates synchronization around spontaneous production rates. Front. Psychol. 9: 458. 29681872 10.3389/fpsyg.2018.00458 PMC5897499

[66] Pfordresher, P.Q. & S. Brown . 2007. Poor‐pitch singing in the absence of “tone deafness.” Music Percept. 25: 95–115.

[67] Klein, G. 2017. Sources of Power: How People Make Decisions. Cambridge, MA: MIT Press.

[68] Taunton, J.E. , M.B. Ryan , D.B. Clement , *et al*. 2002. A retrospective case–control analysis of 2002 running injuries. Br. J. Sports Med. 36: 95–101. 11916889 10.1136/bjsm.36.2.95 PMC1724490

[69] Ferber, R. , B. Noehren , J. Hamill , *et al*. 2010. Competitive female runners with a history of iliotibial band syndrome demonstrate atypical hip and knee kinematics. J. Orthop. Sports Phys. Ther. 40: 52–58. 20118523 10.2519/jospt.2010.3028

[70] Noehren, B. , I. Davis & J. Hamill . 2007. ASB clinical biomechanics award winner 2006 prospective study of the biomechanical factors associated with iliotibial band syndrome. Clin. Biomech. 22: 951–956. 10.1016/j.clinbiomech.2007.07.001 17728030

